# Squalene epoxidase plays a critical role in determining pig meat quality by regulating adipogenesis, myogenesis, and ROS scavengers

**DOI:** 10.1038/s41598-017-16979-x

**Published:** 2017-12-01

**Authors:** Jeongim Ha, Seulgi Kwon, Jung Hye Hwang, Da Hye Park, Tae Wan Kim, Deok Gyeong Kang, Go Eun Yu, Hwa Chun Park, Sang Mi An, Chul Wook Kim

**Affiliations:** 1 Swine Science and Technology Center, Gyeongnam National University of Science & Technology, Jinju, South Korea; 2 Dasan Pig Breeding Co., Namwon, South Korea

## Abstract

In mammals, Squalene epoxidase (SQLE) is an enzyme that converts squalene to 2,3-oxidosqualene, in the early stage of cholesterol generation. Here, we identified single nucleotide polymorphisms (SNPs) in the *SQLE* gene (c.2565 G > T) by RNA Sequencing from the liver tissue of Berkshire pigs. Furthermore, we found that homozygous GG pigs expressed more *SQLE* mRNA than GT heterozygous and TT homozygous pigs in *longissimus dorsi* tissue. Next, we showed that the SNP in the *SQLE* gene was associated with several meat quality traits including backfat thickness, carcass weight, meat colour (yellowness), fat composition, and water-holding capacity. Rates of myogenesis and adipogenesis induced in C2C12 cells and 3T3-L1 cells, respectively, were decreased by *Sqle* knockdown. Additionally, the expression of myogenic marker genes (*Myog*, *Myod*, and *Myh4*) and adipogenic marker genes (*Pparg*, *Cebpa*, and *Adipoq*) was substantially downregulated in cells transfected with *Sqle* siRNA. Moreover, mRNA expression levels of ROS scavengers, which affect meat quality by altering protein oxidation processes, were significantly downregulated by *Sqle* knockdown. Taken together, our results suggest the molecular mechanism by which SNPs in the *SQLE* gene can affect meat quality.

## Introduction

Meat quality, comprising such factors as water-holding capacity, 24-h postmortem pH, cooking loss, drip loss, and shear force, is one of the most important economic traits in the pork industry^1^. As standards of living improve, consumers demand higher quality meat. Accordingly, pig breeders have identified meat quality-associated quantitative trait loci (QTLs) and single nucleotide polymorphisms (SNPs) across the genome to apply marker-associated selection methods for improving meat quality, and to date, thousands of QTLs and SNPs have been identified in this effort^2^. Previously, we identified several SNPs by RNA-Sequencing from the liver tissue of Berkshire pigs. Subsequently, we applied those SNPs to pig breeding procedures to improve meat quality^3–5^, primarily by affecting the production of muscle, the principal component of meat.

Because muscle is composed of myocytes and adipocytes^6^, the development and differentiation of these cell types are considered critical factors in determining meat quality^7,8^. However, few studies have investigated these processes^9,10^. Birth weight is known to be regulated by foetal and prenatal differentiation in myocyte production, and low birth weight in piglets is in turn correlated with decreased rates of growth, and decreased lean percentage at slaughter^11^. Additionally, piglets with low birth weights have fewer muscle fibres than those with higher birth weights. Because the size and number of muscle fibres are inversely correlated, pigs with low birth weights have extremely large muscle fibres that tend to produce low quality meat. Birth weight is controlled by both genetic and maternal factors, and investigation into the effects of specific genes on myogenesis may therefore prove valuable^12^.

Some aspects of the genetic factors involved in myogenesis are well understood. When myogenesis begins, mRNA expression of Pax3 decreases, promoting the mRNA expression of muscle regulatory factors such as Myod, Myog, and Mrf5^13^. Among transcription regulators, Myod and Mrf5 in particular are critical for myoblast determination: mice subjected to Mrf5/Myod double knockout completely lack myoblast and skeletal muscle throughout the body^14^, whereas myoblasts in Myog knockout mice are normal, although such mice lack myotubes^15^. Accordingly, Myog is considered a direct downstream target of Myod and Mrf5 in the muscle network^15^.

Adipocytes comprise the second major component of muscle tissue. CCAAT-enhancer-binding proteins (C/EBPα) can directly force the induction of adipogenic genes, and play a critical role in the development of adipose tissue^16^. Additionally, the peroxisome proliferator-activated receptor gamma (PPARγ) is a well-known transcription factor involved in the differentiation of adipocytes, activating several genes involved in adipocyte lipid storage^17^. To date, no gene has been discovered that promotes adipogenic differentiation in the absence of PPARγ, suggesting that PPARγ is the master regulator for adipogenesis^16^. Leptin is regarded as a late-stage marker of adipocyte differentiation^18^, and is activated by coordination between PPARγ and C/EBPα^19^. Additionally, adiponectin is exclusively expressed and secreted by mature adipocytes and acts as a suitable marker of adipogenesis^20^. While the separate genetic pathways involved in myogenesis and adipogenesis are understood, greater efficiency in control over these processes may be achieved by targeting a single gene that affects both.

In mammals, squalene epoxidase (SQLE) is an enzyme that converts squalene, a 30-carbon linear isoprenoid, to 2,3-oxidosqualene. Squalene synthesis is the first cholesterol-specific step in the pathway, and SQLE catalyses squalene epoxidation. SQLE is an integral ER protein and functions in the presence of NADPH-cytochrome P450 reductase, its electron transfer partner^21^. Although HMG-CoA reductase has been definitely proven to be the primary rate-limiting factor in cholesterol biosynthesis, SQLE has recently been established as a contributing factor, and is also known as a target for hypercholesterolemia therapy in humans^22,23^. In light of its role in cholesterol biosynthesis, SQLE is a prime target for research into genes that control both myogenesis and adipogenesis.

Because of their physiological resemblance to humans, pigs are not only economically valuable sources of popular meat; they are also useful model animals for investigating the genetics of human diseases. Whereas obesity in humans is associated with many diseases, including cardiovascular disease, type 2 diabetes, sleep apnoea, cancer, and osteoarthritis, obesity in pigs is closely associated with carcass weight, average daily weight gain, feeding efficiency, and aspects of meat quality such as fat content and flavour^24,25^. One general characteristic of obesity in both species is the upregulation of cholesterol in the serum, particularly low density lipoprotein cholesterol. Although a close relationship between obesity (or meat quality) and cholesterol level has been reported^26^, the molecules involved in cholesterol biosynthesis and obesity (or meat quality) have not been investigated.

In this study, we identified SNP in the pig *SQLE* gene that may differentially limit rates of cholesterol biosynthesis, and measured the effects of the SQLE genotype on meat quality. Furthermore, we investigated the molecular mechanism by which SQLE can regulate meat quality through myogenesis and adipogenesis. We aimed to generate results that would help to understand the role of SQLE plays in forming muscle tissue in humans (with implications for the treatment of obesity) and in pigs (with implications for the improvement of meat quality).

## Results

### Identification of SNPs in the *SQLE* gene using RNA-Sequencing

The *SQLE* SNP we chose to examine is located on chromosome 4 at 1717 within CDS sequence. The reference nucleotide is G and the variant nucleotide is T. The variant allele is synonymous with the reference allele and does not affect the sequence of amino acids produced (Table 1).

**Table 1:** The information of SNP identified by RNA-Seq in *SQLE* gene.

gene name		SQLE
Locus		Chr4:14339386
RNA-Seq	Reference seq.	G
	Variant seq.	T
	Synonymous	T > T

### Associations between *SQLE* genotypes and meat quality

Data from homozygous GG pigs were compared with a combined pool of data from homozygous TT and heterozygous TG pigs as dominant model. Several meat quality traits (backfat thickness, carcass weight, meat colour yellowness, fat composition, and water-holding capacity) were significantly associated with the *SQLE* genotype (Tables 2 and S3). Homozygous GG pigs had noticeably thicker backfat, and higher carcass weight, fat content, and water-holding capacity than pigs in the combined TT–TG group. Measurements of other meat quality traits (cooking loss, drip loss, chemical composition (protein, collagen, and moisture), shear force, and post mortem pH 24 h) did not associate by *SQLE* genotype (Table S3).

**Table 2:** The association between genotype of *SQLE* and meat quality traits.

	Model	Dominant			
	Genotype	TT + GT (n = 38)		GG (n = 350)	
		Mean	STD	Mean	STD
Traits	Backfat thickness	22.606^#^	4.054	26.390^#^	4.212
	Carcass weight (kg)	84.727*	3.923	86.455*	4.820
	Meat color CIE b	2.439*	0.944	2.862*	1.097
	Chemical composition (%) fat	2.493^#^	0.737	2.879^#^	1.134
	Water-holding capacity (%)	57.305*	2.045	58.144*	2.684

CIE b respresent the meat color yellowness. *^,#^Value is significantly different (^#^ *P* < 0.01 *P < 0.05) in the genotypes.

### Effect of the *SQLE* gene on myogenesis and adipogenesis

**Figure 1:**
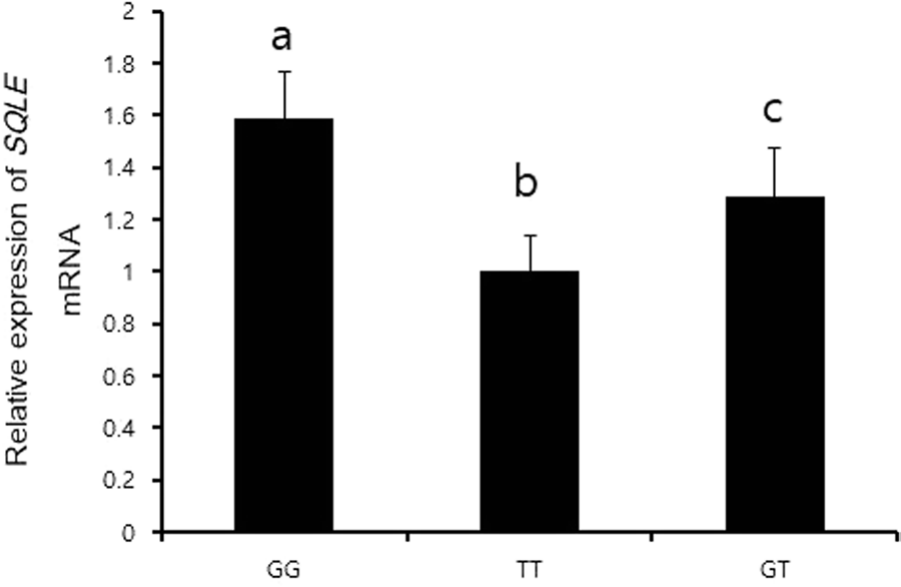
mRNA expression of *SQLE* according to genotype of *SQLE*. The *longissimus dorsi* tissue of four pigs from each genotype (GG, GT, and TT) was prepared. mRNA expression of *SQLE* was analysed by RT-PCR. *PPIA* was used as an internal control gene. Band intensity was measured using ImageJ. The fold change of *SQLE* mRNA expression levels was calculated by comparing expression levels of the genotype with the lowest expression level (homozygous TT) versus those of the other genotypes. Data was expressed as mean ± SD. The significant differences (*P* < 0.05) were shown as different letter analysed between groups.

**Figure 2:**
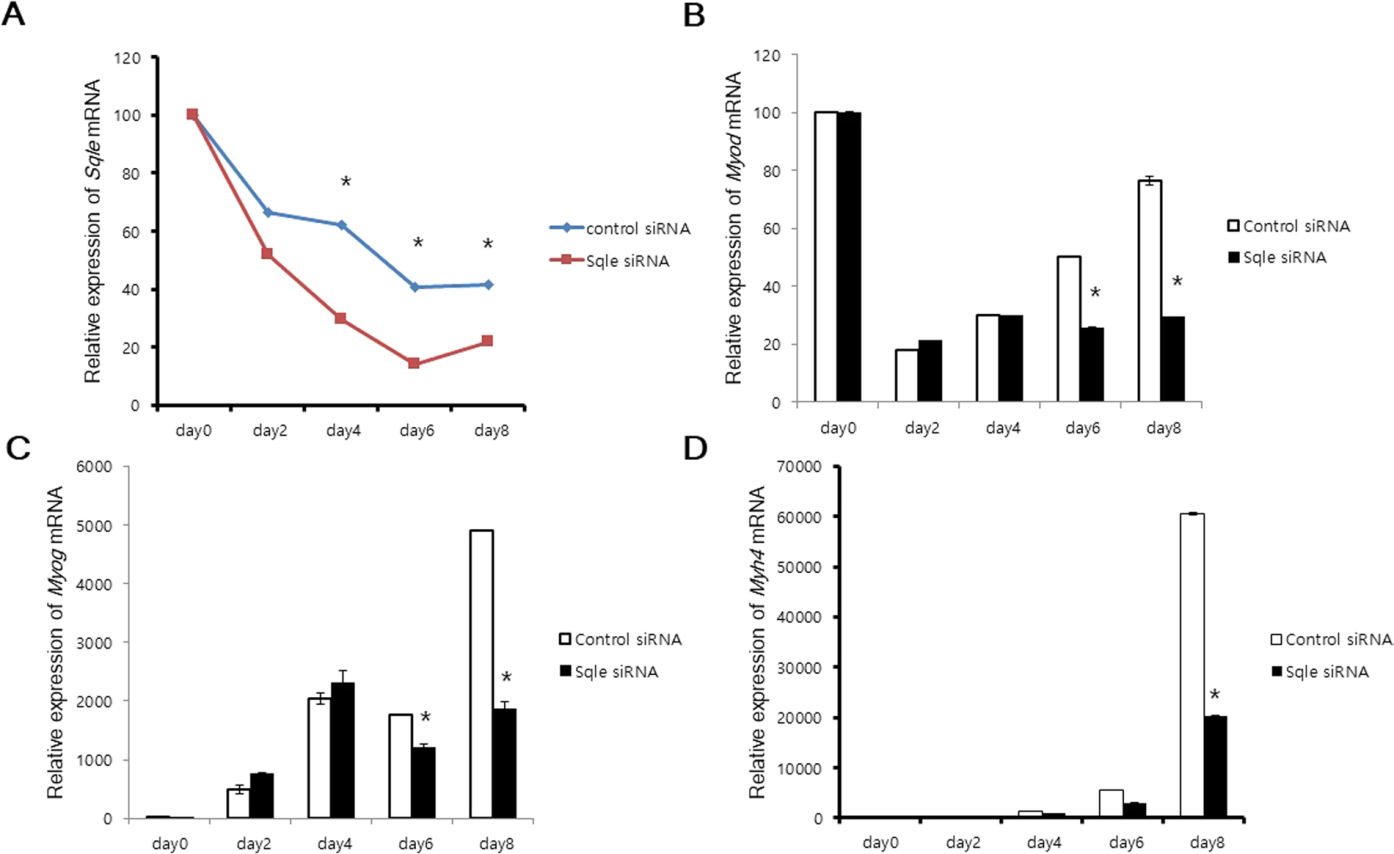
The effect of *Sqle* on myogenesis. (**A**–**D**) C2C12 cells were cultured in DMEM with 10% FBS in the presence of Pen Strep. Cells were transfected with control and *Sqle* siRNA using RNAiMAX when their confluency reached 50%. After 24 h recovery, cells began to induce myogenic differentiation under exposure to a treatment of 2% heat-inactivated horse serum. Cells were prepared on days 2, 4, 6, and 8 after the beginning of differentiation and were subjected to RT-qPCR. Gene expression levels (A;*Sqle*, B;*Myod*, C;*Myog*, D;*Myh4*) were analysed using the 2^−∆∆^Cq method. Gapdh was used as an internal control gene. Fold change was calculated by dividing expression levels in the experimental group by those of control group. All experiments were done at least three repeats. Data was expressed as mean ± SD. *P < 0.05 versus control siRNA transfection.

**Figure 3:**
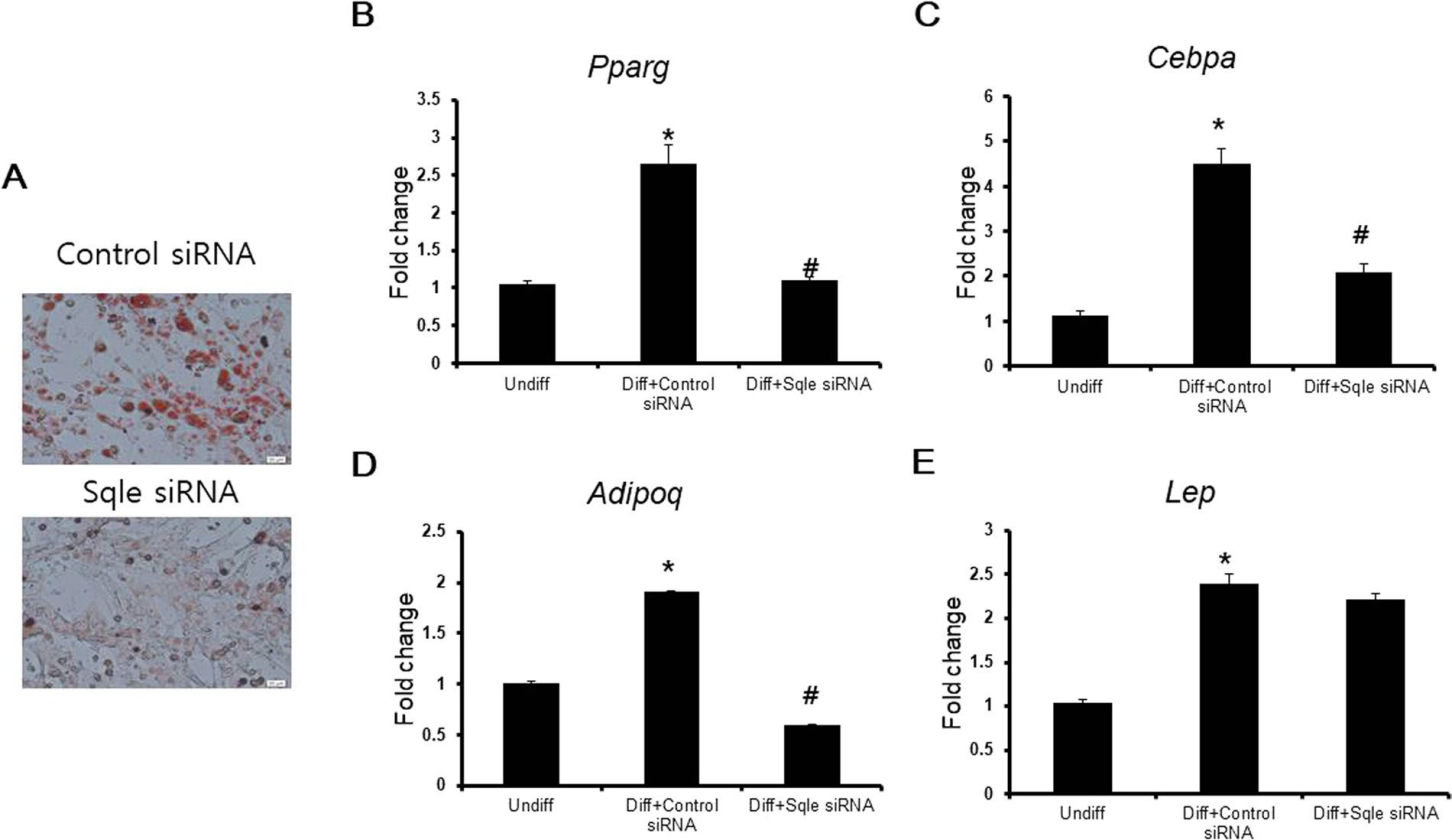
The effect of *Sqle* on adipogenesis. (**A**) 3T3-L1 cells were seeded on 24-well plate. After 24 h, cells were transfected with control and *Sqle* siRNA. When the confluency of cells reached 70%, at which point 3T3-L1 cells were differentiated in adipogenic differentiation media containing DMEM supplemented with 10 µg/mL insulin, 0.5 mM 3-isobutyl-1-methylxanthine (IBM-X), and 1 μM dexamethasone. Cells were fully differentiated into adipocytes after 7 d, at which point cells were fixed in 10% (v/v) formaldehyde in PBS and stained with Oil Red O solution. Stained cells were observed under a microscope. (**B**–**D**) Cells were seeded on 6-well plate. After 24 h, cells were transfected with control and *Sqle* siRNA. The adipogenic differentiation was induced as described above. The RNA from undifferentiated cells and differentiated cells transfected with control and *Sqle* siRNA was prepared and subjected to RT-qPCR. Gene expression levels (B;*Pparg*, C;*Cebpa*, D;*Adipoq*, E;*Lep*) were analysed using the 2^−∆∆^Cq method. *Gapdh* was used as an internal control gene. Fold change was calculated by dividing expression levels in the experimental group by those of the undifferentiated group. All experiments were done at least three repeats. The significant differences were shown as *P < 0.05 versus undifferentiated cells and ^#^ *P* < 0.05 versus control siRNA transfection.

Since synonymous SNP can affect its mRNA structure and stability, we investigated whether the presence of the SNP in the *SQLE* gene would lead to change in its mRNA expression. Heterozygous TG and homozygous TT pigs expressed significantly less *SQLE* mRNA than homozygous GG pigs (Fig. 1). Because the SNP in the *SQLE* gene affected its mRNA expression, we used an siRNA transfection system to mimic TG-TT pigs, in which *SQLE* mRNA expression was down regulated with respect to that in GG pigs. We also aimed to reveal the effect of SQLE on myogenesis and adipogenesis. Since myocytes and adipocytes are major component of muscle, the development and differentiation of these cells are important factors in regulating meat quality. *Sqle* mRNA expression gradually decreased during myogenic differentiation (Fig. 2A), and *Sqle* siRNA continued functioning until myogenesis was complete. The efficiency of knockdown was approximately 50% (Fig. 2A). Expression levels of the myogenic marker genes *Myod*, *Myog*, and *Myh4* were significantly decreased by *Sqle* knockdown (Fig. 2B–D). Additionally, lipid droplet accumulation in adipocytes was significantly decreased by *Sqle* knockdown (Fig. 3A). Expression levels of adipogenic marker genes, such as *Adipoq*, *Pparg*, and *Cebpa*, were significantly decreased by *Sqle* siRNA transfection; however, the mRNA expression levels of *Lep* did not change.

### Effects of *Sqle* on reactive oxygen species (ROS) scavengers

**Figure 4:**
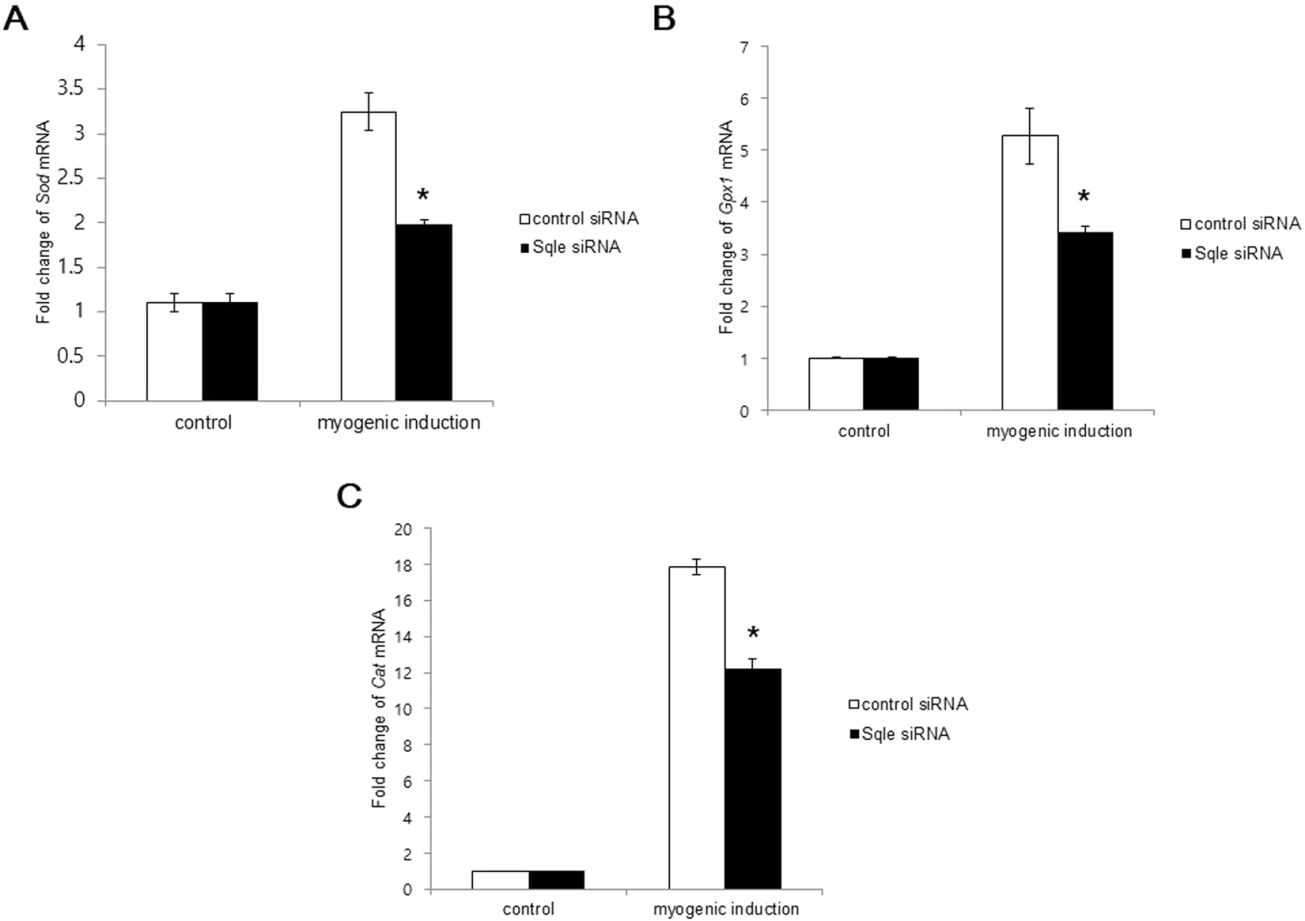
The effect of *Sqle* on ROS scavengers. (**A**–**C**) C2C12 cells were cultured and transfected as mentioned in Fig. 2. Cells were prepared on 2 d of myogenic induction and subjected to RT-qPCR. Gene expression levels (A;*Sod*, B;*Gpx1*, C;*Cat*) were analysed using the 2^−∆∆^Cq method. *Gapdh* was used as an internal control gene. The fold change of ROS scavengers was calculated by dividing expression levels in the myogenic group by those of control group. The experiment was done at least three times repeats. The significant differences were shown as *P < 0.05 versus control.

In order to elucidate the effect of *Sqle* on the activities of ROS scavengers such as superoxide dismutase (Sod), catalase (Cat), and glutathione peroxidase 1 (GPx1), we used C2C12 cells transfected with control and *Sqle* siRNA. Myogenic induction for 2 d promoted the induction of ROS scavengers. However, *Sqle* knockdown significantly repressed the induction of ROS scavengers (Fig. 4A–C). Because oxidative stress has been known to affect protein oxidation, which in turn affects meat quality, *Sqle* might play a critical role in determining meat quality via protein oxidation by regulating ROS scavenger induction.

In conclusion, variants in the *SQLE* gene had significant associations with pig meat quality. The GG genotype in the *SQLE* gene is preferable for breeding. We inferred that SQLE plays a critical role in determining the amount of meat and fat on basis of siRNA system data on myogenesis and adipogenesis. Furthermore, in this regard, SQLE might be closely associated with human diseases such as obesity, cardiovascular disease, and type 2 diabetes.

## Discussion

In the present study, to uncover the SNP markers associated with meat quality, we performed RNA-Sequencing using the liver tissue of Berkshire pigs. As a result, SNP in the *SQLE* gene have been identified and shown to be closely associated with meat quality traits such as backfat thickness, carcass weight, meat colour, fat composition, and water-holding capacity. The results of the present study support our expectation that *SQLE* SNPs differentially affect aspects of meat quality; however, the effects of *SQLE* SNPs on meat quality were indirect, rather than direct. Coding region SNPs are divided into two groups: synonymous SNPs and non-synonymous SNPs. Generally, non-synonymous SNPs, which cause changes in amino acid sequences, have a substantial effect on enzyme activity^27^. Although synonymous SNPs do not affect amino acid sequences, they affect several cellular pathways, and regulate processes relating to the structure and stability of mRNA, kinetics of translation, and alternate splicing^28^. The SNP we identified in the *SQLE* gene in this study was defined as synonymous. The heterozygous TG and homozygous TT pigs expressed less *SQLE* mRNA than the homozygous GG pigs, suggesting that the *SQLE* SNP may affect mRNA stability nevertheless. In this regard, we used the siRNA system to reveal the molecular mechanism by which meat quality could be regulated via mimicry of the down regulation of mRNA expression in the TG-TT pigs.

Since muscle and fat are major component of meat, we performed myogenesis and adipogenesis using SQLE siRNA. As the homology of the porcine and murine *SQLE* genes would be approximately 82%, we used murine cell lines such as C2C12 and 3T3-L1, which are well-developed model systems for the differentiation of muscle and fat, respectively. Both myogenesis and adipogenesis were significantly suppressed by *Sqle* siRNA transfection, as were myogenic (*Myod*, *Myog*, *Myh4*) and adipogenic (*Aipoq*, *Pparg*, *Cebpa*) marker genes, suggesting that *Sqle* may regulate the myogenic marker gene at a transcriptional level, functioning as an upstream signalling molecule. In addition, squalene or intermediates produced by SQLE may also act as signalling molecules. *Myod* mRNA expression decreased by approximately 50% (Fig. 2), the same decrease in efficiency as that caused by *Sqle* siRNA knockdown. However, the proportional decrease of *Myog* expression by *Sqle* knockdown was greater than that of *Myod*. Because Myod is an initial regulating factor in myogenesis and Myog is a direct downstream target for Myod, this result suggests that Myog is controlled not only by Myod but also by other regulators independent of Sqle. Furthermore, *Myh4* expression may be modulated by Sqle in a direct pathway, and mediated by other molecules in indirect pathways.

Although both PPARγ and C/EBPα regulate the expression of adipocyte-specific genes, the expression of adiponectin but not of leptin was affected by *Sqle* siRNA transfection, which suppressed induction of both PPARγ and C/EBPα. Although adiponectin is primarily regulated by the PPARγ transcription factor, leptin is regulated by several transcription factors, including hypoxia-induced factor 1, SP-1, and STAT3^29–32^. We infer from this result that the effect of Sqle knockdown on adiponectin was substantial, whereas the effect on leptin was marginal. As Sqle knockdown directly controlled adipogenesis *in vitro*, fat content in pigs was significantly associated with the genotype of the *SQLE* gene. The transcriptional regulator for myogenesis usually controls the balance between intramuscular adipogenesis and myogenesis^33^; however, our results indicate that Sqle plays a role in stimulating both adipogenesis and myogenesis.

Our hypothesis that there was a close relationship between growth performance and *SQLE* genotype is supported by our results, which showed that the TT-GT pigs had significantly lower carcass weights than pigs in the homozygous GG pigs. Because pigs in the TT–GT group expressed lower levels of *SQLE* mRNA than homozygous GG pigs, the effects of siRNA-induced myogenesis were significantly reduced. This result implies that the significant decrease in carcass weight in the TT-GT pigs was caused by decreased *SQLE* mRNA expression.

An increase in intracellular ROS promotes protein oxidation. Muscle protein oxidation leads to protein fragmentation and aggregation, which in turn result in poor meat quality. Among meat quality traits, water-holding capacity is particularly susceptible to protein oxidation products such as oxidized actin and oxidized aggregates^34^. ROS scavengers such as superoxide dismutase (Sod), catalase (Cat), and glutathione peroxidase 1 (GPx1) might prevent protein oxidation in slaughtered meat. Therefore, we assessed whether Sqle can affect the mRNA expression of ROS scavengers. When we used the siRNA system, *Sqle* knockdown significantly decreased the mRNA expression of ROS scavengers. Thus, Sqle might directly regulate ROS scavenger induction as an upstream molecule. The specific mechanism by which SQLE regulates ROS scavengers should a focus for future research.

Our approach, might lead to only indirect estimations of the relation between meat quality traits and the effects of Sqle on myogenesis and adipogenesis, because this relationship was analysed in pigs by SNP and the determination of effects on myogenesis and adipogenesis was conducted using the siRNA system. Although we used the siRNA system to mimic TT-GT type groups which expressed lesser *SQLE* mRNA than did the homozygous GG type, to ensure the results are directly transferable the point mutation construct for the *Sqle* should be administrated into myogenesis and adipogenesis in future studies.

To convince the results from meat quality traits according to SNP in *SQLE* gene, we also performed another round of experiments with reproducibility tests for pig meat quality and genotype analysis. The experiment was conducted using 30 pigs. The homozygous GG pigs (n = 21) were shown to have significantly higher water-holding capacity than did TT-GT pigs with the same tendency in the primary experiment (Table S4).

We identified a SNP in the *SQLE* gene and detected an association between the *SQLE* genotype and meat quality in pigs, where the *SQLE* siRNA affected myogenesis, adipogenesis, and ROS scavenger induction. In conclusion, our results suggest that the molecular mechanism by which SQLE might affect meat quality.

## Methods

### Animal treatment and ethics statement

A total of 418 Berkshire pigs were used in this study, and were reared under identical conditions by Dasan Genetics (Namwon, Korea). Pigs were slaughtered in 10 batches at a body weight of 110 kg. At the time of slaughter, *longissimus dorsi* tissue samples were collected and were prepared to be analysed for measures of meat quality(n = 418), and liver tissue samples were collected and were prepared for RNA sequencing (n = 3). Whole blood was collected from each animal and was prepared for genotype analysis (n = 418). In the Republic of Korea, approval for experiments involving livestock is not required. However, the pigs used in this study received care according to the guidelines proposed by the Animal Care and Use Committee of Gyeongnam National University of Science and Technology (ACUC of GNTECH), and according to the Korean Animal Protection Act and related laws.

### RNA sequencing

To identify SNPs, RNA was sequenced using total RNA from the liver tissues of 3 Berkshire pigs. Total RNA was prepared using TRI-Reagent (Molecular Research Center, Cincinnati, OH, USA) according to instructions specified by the manufacturer. mRNA was purified using an RNA sequencing sample preparation kit (Illumina, Inc., San Diego, CA, USA). SNPs were detected using a GAII analyzer (Illumina, Inc., San Diego, CA, USA) according to methods described in a previous study^4^. The analysis included total trimmed reads, which were assembled and mapped to the annotated pig transcriptome assembly in the UniGene database.

### Genotyping of SNPs in the *SM* gene

Genomic DNA (gDNA) from the blood of 418 Berkshire pigs was extracted using the Wizard Genomic DNA Purification Kit (Promega, Madison, WI, USA) following instructions specified by the manufacture. To analyze the genotyping of the *SQLE* gene, a gDNA assay was carried out in a VeraCode GoldenGate (Illumina, Inc., San Diego, CA, USA) using specific oligonucleotides (Table S1). Three genotypes were identified using these methods: homozygous GG, heterozygous TG, and homozygous TT.

### Analyses of meat quality traits

Traits contributing to meat quality were measured using methods described in a previous study^3^. Briefly, backfat thickness was measured at a distance of three-quarters along the muscle towards the belly. Water-holding capacity was calculated as a percentage of water lost during centrifugation 3 d postmortem. *Longissimus dorsi* tissue samples were cut in water, and a portable pH meter was used to measure pH 24 h postmortem (pH24 h). Meat colour was measured using a colorimeter after blooming for 15 min under a light source (Minolta, CR-400, Tokyo, Japan). Cooking loss was calculated by measuring differences in the weights of samples before and after cooking for 40 min at 70 °C. Drip loss was calculated by weighing tissue samples before and after storage at 4 °C for 24 h. The chemical composition of samples (protein, fat, collagen, and moisture) was determined according to methods proposed by the Association of Official Agricultural Chemists^35^. The Warner-Bratzler test, in which shearing follows the direction of the fibres, was used to measure shear force in the muscle tissues.

### RT-PCR

RNA (3 µg) was extracted from the *longissimus dorsi* tissue of four pigs from each genotype (with the genotypes homozygous TT, heterozygous GT, and homozygous GG) using an RNAeasy mini kit (Qiagen, Valencia, CA, USA). Reverse transcription into cDNA was carried out using Invitrogen Superscript II (Thermo Fisher Scientific, Inc., Waltham, MA, USA) with a total reaction volume of 20 µL. mRNA expression of the *SQLE* gene was measured using primers annealed specifically to the *SQLE* gene (Table S2). PCR reaction for *SQLE* gene specific primers was carried out for 32 cycles of 20 s at 95 °C, 20 s at 60 °C, and 20 s at 72 °C. Peptidylprolyp isomerase A (*PPIA*) was used as an internal control gene and subjected to PCR reaction for 20 cycles of same procedure as done for *SQLE*. PCR products were separated in a 2% agarose gel and were visually inspected under a UV spectrophotometer. Band intensity was measured using ImageJ. The fold change of *SQLE* mRNA expression levels was calculated by comparing expression levels of the genotype with the lowest expression level (homozygous TT) with those of the other genotypes.

### Real-time reverse-transcription quantitative PCR (RT-qPCR)

Prior to real-time qPCR, reverse transcription of samples was carried out using the methods described above. Real-time qPCR was carried out using a Rotor Gene-Q thermocycler (Qiagen, Valencia, CA, USA). In each reaction, 1 µL cDNA was added into 10 µL “mastermix”, comprising 5 µL Rotor Gene SYBR Green PCR MasterMix, 1 µL forward and 1 µL reverse primers (Table S2), and 3 µL H_2_O. PCR was carried out for 40 cycles of 5 s at 94 °C and 10 s at 60 °C. Glyceraldehyde 3-phosphate dehydrogenase (*Gapdh*) was used as an internal control gene. Amplification specificity was confirmed by melting curve analysis at temperatures of 70–95 °C for 5 s. Gene expression levels were analysed using the 2^−∆∆^Cq method. Fold change was calculated by dividing expression levels in the experimental group by those of the group with the lowest expression level. All experiments were carried out according to the MIQE guidelines^36^. Student’s *t*-tests and ANOVA were used to detect differences in expression levels between groups.

### Myogenic differentiation

To detect the effects of SNPs in the *SM* gene on myogenesis and adipogenesis, we used *Sqle* siRNA to mimic the effect of a genotype with downregulated mRNA expression. We used C2C12 myoblast cells to examine the effects of *Sqle* on myogenesis. C2C12 myoblast cells were purchased from ATCC (Manassas, VA, USA) and were initially cultured in growth media containing Dulbecco’s Modified Eagle Medium (DMEM) with 10% fetal bovine serum (FBS) and Pen Strep (100 U mL^−1^ penicillin and 100 µg mL^−1^ streptomycin). Cell culture media were changed every 2 d. Cells were cultured at 37 °C in a humidified incubator (5% CO_2_, 95% air). On the second day of culturing, when cultures reached 50% cell confluence, C2C12 cells were transfected with control siRNA (Ambion, 4390843) and *Sqle* siRNA (Ambion, s74372) using the Invitrogen RNAiMAX transfection reagent (all purchased from Thermo Fisher Scientific, Inc., Waltham, MA, USA) according to instructions specified by the manufacturer. After 24 h recovery, C2C12 cells began to differentiate under exposure to a treatment of Gibco 2% heat-inactivated horse serum (Thermo Fisher Scientific, Inc., Waltham, MA, USA). Cells were prepared on days 2, 4, 6, and 8 after the beginning of differentiation and were subjected to RT-qPCR.

### Adipogenic differentiation

We used 3T3-L1 pre-adipocytes, purchased from ATCC (product ATCC^®^ CL-173^TM^, Manassas, VA, USA), to examine the effects of *Sqle* on adipogenesis. 3T3-L1 cells were cultured in a growth medium comprising DMEM, 10% bovine calf serum, and Pen Strep (100 U mL^−1^ penicillin and 100 µg mL^−1^). Cells were cultured at 37 °C in a humidified incubator (5% CO_2_, 95% air). 3T3-L1 cells were seeded on a six-well plate for RT-qPCR and a 24-well plate for Oil Red O staining. After 24 h, 3T3-L1 cells were transfected with control and *Sqle* siRNA using the procedures described above for C2C12 cells. The medium was changed every 2 days until cells had reached 70% confluence, at which point 3T3-L1 cells were differentiated in adipogenic differentiation media containing DMEM supplemented with 10 µg/mL insulin, 0.5 mM 3-isobutyl-1-methylxanthine (IBM-X), and 1 μM dexamethasone (all purchased from Sigma-Aldrich, St. Louis, MO, USA). 3T3-L1 adipocytes that were fully differentiated 7 d after culturing were subjected to RT-qPCR and Oil Red O staining. Fully differentiated adipocytes contained lipid droplets that accumulated lipid bodies, and were visible after Oil Red O staining.

### Determination of mRNA expression of ROS scavengers by RT-qPCR

C2C12 cells were cultured and transfected with siRNA as described above. Cells were prepared on 2 d of myogenic induction and subjected to RT-qPCR. The fold change of ROS scavengers was calculated by dividing expression levels in the myogenic group by those of control group. The experiment was done at least three times repeats. The significant differences were analysed by Student *t*-test.

### Statistical analysis

To analyse significant differences between meat quality traits and genotype in the *SQLE* gene, we used IBS SPSS Statistic 23. The statistical significance in the dominant model was assessed using Student-*t* tests with differences being considered significant at *P* < 0.05. When the results were corroborated by the homogeneity of variance, T values and significance were assessed. A general linear model was used to detect differences between different genotypes and measurements of meat quality traits, using SAS software (ver. 9.1.3; SAS Institute, Cary, NC, USA). The linear model had the form *y*_*ijk*_ = *µ* + *G*_*i*_ + *S*_*j*_ + *P* + *e*_*ijk*_, where *y*_*ijk*_ was the phenotypic value of the target trait, µ was the general mean, *G*_*i*_ was the fixed effect of genotype *i*, *S*_*j*_ was the fixed effect of gender *j, P* was the fixed effect of slaughter period, and *e*_*ijk*_ was the random error term. SNPs were selected for statistical analysis according to call rate (>90%), minor allele frequency (MAF > 0.01), and conformance to proportions of Hardy–Weinberg equilibrium (HWE; *P* > 0.05). All PCR experiments were repeated at least three times and were presented as the mean ± SD. The significance of the level of mRNA expression according to genotype in the *SQLE* gene, was analysed using ANOVA with differences being considered at *P* < 0.05. The other results were analysed using Student’s-*t* tests with differences being considered significant at *P* < 0.05.

## Electronic supplementary material

supplementary table

## References

[1] Malek M (2001). A molecular genome scan analysis to identify chromosomal regions influencing economic traits in the pig. II. Meat and muscle composition. Mammalian genome: official journal of the International Mammalian Genome Society.

[2] Ernst CW, Steibel JP (2013). Molecular advances in QTL discovery and application in pig breeding. Trends in genetics: TIG.

[3] Cho HR (2015). Single-Nucleotide Polymorphisms in Pig EPHX1 Gene are Associated with Pork Quality Traits. Animal biotechnology.

[4] Jung WY (2012). RNA-Seq approach for genetic improvement of meat quality in pig and evolutionary insight into the substrate specificity of animal carbonyl reductases. PloS one.

[5] Jo, J. L. *et al*. Association between a non-synonymous HSD17B4 single nucleotide polymorphism and meat-quality traits in Berkshire pigs. *Genetics and molecular research: GMR* **15**, 10.4238/gmr15048970 (2016). 10.4238/gmr15048970 27819726

[6] Du M (2011). Fetal muscle development, mesenchymal multipotent cell differentiation, and associated signaling pathways. Journal of animal science.

[7] Hausman GJ, Poulos SP, Pringle TD, Azain MJ (2008). The influence of thiazolidinediones on adipogenesis *in vitro* and *in vivo*: potential modifiers of intramuscular adipose tissue deposition in meat animals. Journal of animal science.

[8] Kokta TA, Dodson MV, Gertler A, Hill RA (2004). Intercellular signaling between adipose tissue and muscle tissue. Domestic animal endocrinology.

[9] Li FN (2010). Chloride intracellular channel 5 modulates adipocyte accumulation in skeletal muscle by inhibiting preadipocyte differentiation. Journal of cellular biochemistry.

[10] Zhao C (2016). MAT2B promotes adipogenesis by modulating SAMe levels and activating AKT/ERK pathway during porcine intramuscular preadipocyte differentiation. Experimental cell research.

[11] Du M, Wang B, Fu X, Yang Q, Zhu MJ (2015). Fetal programming in meat production. Meat science.

[12] Rehfeldt C, Kuhn G (2006). Consequences of birth weight for postnatal growth performance and carcass quality in pigs as related to myogenesis. Journal of animal science.

[13] Yusuf F, Brand-Saberi B (2006). The eventful somite: patterning, fate determination and cell division in the somite. Anatomy and embryology.

[14] Rudnicki MA (1993). MyoD or Myf-5 is required for the formation of skeletal muscle. Cell.

[15] Hasty P (1993). Muscle deficiency and neonatal death in mice with a targeted mutation in the myogenin gene. Nature.

[16] Farmer SR (2006). Transcriptional control of adipocyte formation. Cell metabolism.

[17] Tontonoz P, Hu E, Spiegelman BM (1994). Stimulation of adipogenesis in fibroblasts by PPAR gamma 2, a lipid-activated transcription factor. Cell.

[18] Gregoire FM, Smas CM, Sul HS (1998). Understanding adipocyte differentiation. Physiological reviews.

[19] Hollenberg AN (1997). Functional antagonism between CCAAT/Enhancer binding protein-alpha and peroxisome proliferator-activated receptor-gamma on the leptin promoter. The Journal of biological chemistry.

[20] Kiess W (2008). *Adipocytes and adipose tissue*. Best practice & research. Clinical endocrinology & metabolism.

[21] Sakakibara J, Ono T (1994). [Squalene epoxidase: another rate limiting enzyme in cholesterol biosynthesis]. Tanpakushitsu kakusan koso. Protein, nucleic acid, enzyme.

[22] Belter A (2011). Squalene monooxygenase - a target for hypercholesterolemic therapy. Biological chemistry.

[23] Sharpe LJ, Brown AJ (2013). Controlling cholesterol synthesis beyond 3-hydroxy-3-methylglutaryl-CoA reductase (HMGCR). The Journal of biological chemistry.

[24] Quispe R, Martin SS, Jones SR (2016). Triglycerides to high-density lipoprotein-cholesterol ratio, glycemic control and cardiovascular risk in obese patients with type 2diabetes. Current opinion in endocrinology, diabetes, and obesity.

[25] Rauw WM (2012). The relationship between feed intake behaviour with intramuscular fat, cholesterol and fatty acid composition in pork. Journal of animal breeding and genetics = Zeitschrift fur Tierzuchtung und Zuchtungsbiologie.

[26] Bijari B (2015). The relationship between serum lipids and obesity among elementary school in Birjand: a case control study. Journal of research in health sciences.

[27] Teng S, Michonova-Alexova E, Alexov E (2008). Approaches and resources for prediction of the effects of non-synonymous single nucleotide polymorphism on protein function and interactions. Current pharmaceutical biotechnology.

[28] Shastry BS (2009). SNPs: impact on gene function and phenotype. Methods Mol Biol.

[29] Grosfeld A (2002). Hypoxia-inducible factor 1 transactivates the human leptin gene promoter. The Journal of biological chemistry.

[30] Moreno-Aliaga MJ (2007). Sp1-mediated transcription is involved in the induction of leptin by insulin-stimulated glucose metabolism. Journal of molecular endocrinology.

[31] Munzberg H, Huo L, Nillni EA, Hollenberg AN, Bjorbaek C (2003). Role of signal transducer and activator of transcription 3 in regulation of hypothalamic proopiomelanocortin gene expression by leptin. Endocrinology.

[32] Iwaki M (2003). Induction of adiponectin, a fat-derived antidiabetic and antiatherogenic factor, by nuclear receptors. Diabetes.

[33] Zhao X (2011). Comparative analyses by sequencing of transcriptomes during skeletal muscle development between pig breeds differing in muscle growth rate and fatness. PloS one.

[34] Traore S (2012). Higher drip loss is associated with protein oxidation. Meat science.

[35] AOAC International. volumes (loose-leaf) (AOAC International, Arlington, Va., 1995).

[36] Bustin SA (2009). The MIQE guidelines: minimum information for publication of quantitative real-time PCR experiments. Clinical chemistry.

