# Supplementary material for: Squalene epoxidase plays a critical role in determining pig meat quality by regulating adipogenesis, myogenesis, and ROS scavengers
Source: Sci Rep. 2017 Dec 1;7:16740. doi: 10.1038/s41598-017-16979-x (PMC5711910; doi:10.1038/s41598-017-16979-x)
Supplement: Supplementary file 1 — supplementary table [file 41598_2017_16979_MOESM1_ESM.pdf]

Squalene epoxidase plays a critical role in determining pig meat quality by regulating adipogenesis, myogenesis, and ROS scavengers

Jeongim Ha[1], Seulgi Kwon[1], Jung Hye Hwang[1], Da Hye Park[1], Tae Wan Kim[1], Deok Gyeong Kang[1], Go Eun Yu[1], Hwa Chun Park[3], Sang Mi An[1], and Chul Wook Kim[1*]

Table S1. Allele-specific oligonucleotide used for genotyping of SNP in SQLE gene

Oligonucleotied	Sequence
Allele-specific Oligo 1	5'-ACTTCGTCAGTAACGGACGAAAAGCAACAGTCATTCCTCCTCCA-3'
Allele-specific Oligo 2	5'-GAGTCGAGGTCATATCGTGAAAAGCAACAGTCATTCCTCCTCCC-3'
Allele-specific Oligo 3	5'-TGAGAGGATGCCTCATGTTATCTCAGATACCGAGACTTGGGCCGTCTGCCTATAGTGAGTC-3'

Table S2. Oligonucleotides used in this study

names		Nucleotide sequences (5′-3′)
SQLE (pig)	F	GCTTCTTCTGGGCCAAATCC
	R	AAAGCAGAGCCAAGTACACC
PPIA (pig)	F	CACAAACGGTTCCCAGTTTT
	R	TGTCCACAGTCAGCAATGGT
Sqle (mouse)	F	CTGGGCCTTGGAGATACAGT
	R	TGCTTTCCGGAGACTCATGA
Gapdh (mouse)	F	AGTGTTTCCTCGTCCCGTAG
	R	ATGTTAGTGGGGTCTCGCTC
Adipoq (mouse)	F	TGTAGGATTGTCAGTGGATCTG
	R	GCTCTTCAGTTGTAGTAACGTCAT
Lep (mouse)	F	CCAGGATGACACCAAAACCC
	R	TGCAGCACATTTTGGGAAGG
Pparg (mouse)	F	CCGCCGCCTCAGATTTGA
	R	TGTCAAAGGAATGCGAGTGG
Cebpa (mouse)	F	CGGTGACTTTGACTACCCGG
	R	GGGGCTCTTGTTTGATCACC
Myog (mouse)	F	CCAGTGAATGCAACTCCCAC
	R	GCATGGTTTCGTCTGGGAAG
Myod (mouse)	F	GCCTTCTACGCACCTGGACC
	R	GGCGGTGTCGTAGCCATTCT
Myh4 (mouse)	F	CTTTGACAAGGTCCTGGCAGAATG
	R	TGGATGTGCTTCCCTCCCTCAG
Sod1 (mouse)	F	GGAAGCATGGCGATGAAAGC
	R	AGCCTTGTGTATTGTCCCCA
Cat (mouse)	F	ACACTTTGACAGAGAGCGGA
	R	GTGGAGAATCGAACGGCAAT
Gpx1 (mouse)	F	GGTTTCCCGTGCAATCAGTT
	R	CAGGAAGGTAAAGAGCGGGT

Table S3. Analysis of association between *SQLE* SNP and pork meat quality traits

SNV1 chromosomal location			Chr4:g14339386			
Model			Dominant			
Genotype			TT+TG (n=38)		GG (n=350)	
			Mean	SE	Mean	SE
Traits	Backfat thickness		22.606 #	4.054	26.390 #	4.212
	Carcass weight (kg)		84.727 *	3.923	86.455 *	4.820
	Meat color	CIE L	48.491	2.927	48.619	2.927
		CIE a	5.862	0.857	6.178	1.070
		CIE b	2.439 *	0.944	2.862 *	1.097
	Cooking loss (%)		28.233	2.903	27.309	3.625
	Drip loss (%)		4.468	1.596	4.547	1.961
	Chemical composition (%)	Protein	23.892	0.591	23.806	0.743
		Fat	2.493 #	0.737	2.879 #	1.134
		Collagen	0.891	0.095	0.891	0.138
		Moisture	75.581	0.813	75.508	0.877
	Warner-Bratzler shear force		2.792	0.452	2.917	0.703
	Water-holding capacity (%)		57.305 *	2.045	58.144 *	2.684
	pH24hr		5.869	0.187	5.816	0.219

*,# Value is significantly different (#P < 0.01 *P < 0.05) between two groups

CIE L, a and b respectively represent the meat color lightness, redness and yellowness.

Table S4. Reproducibility experiment for the association between *SQLE* SNP and pork meat quality traits

SNV1 chromosomal location			Chr4:g14339386			
Model			Dominant			
Genotype			TT+GT (n=9)		GG (n=21)	
			Mean	SE	Mean	SE
Traits	Meat color	CIE L	48.721	6.197	48.755	5.540
		CIE a	7.011	2.223	7.640	1.693
		CIE b	2.114	0.908	2.425	1.200
	Cooking loss (%)		37.223	1.811	36.940	1.707
	Drip loss (%)		7.153	4.502	6.371	4.004
	Warner-Bratzler shear force		4.992	1.480	4.581	1.908
	Water-holding capacity (%)		60.926 *	1.254	62.916 *	2.081
	pH24hr		5.721	0.202	5.742	0.201

*,# Value is significantly different ($^{\#}P < 0.01$ $^{*}P < 0.05$) between two groups

CIE L, a and b respectively represent the meat color lightness, redness and yellowness.
